# Synergistic Effect of Fluconazole and Calcium Channel Blockers against Resistant *Candida albicans*

**DOI:** 10.1371/journal.pone.0150859

**Published:** 2016-03-17

**Authors:** Shuyuan Liu, Longtao Yue, Wenrui Gu, Xiuyun Li, Liuping Zhang, Shujuan Sun

**Affiliations:** 1 School of Pharmaceutical Sciences, Shandong University, Jinan, 250012, Shandong Province, People’s Republic of China; 2 Department of Pharmacy, Ordos Central Hospital, Ordos, 017000, Inner Mongolia, People’s Republic of China; 3 Translational Medicine Research Centre, Qianfoshan Hospital Affiliated to Shandong University, Jinan, 250014, Shandong Province, People’s Republic of China; 4 School of Pharmaceutical Sciences, Taishan Medical University, taian, 271000, Shandong Province, People’s Republic of China; 5 Department of Pharmacy, Qianfoshan Hospital Affiliated to Shandong University, Jinan, 250014, Shandong Province, People’s Republic of China; California Department of Public Health, UNITED STATES

## Abstract

Candidiasis has increased significantly recently that threatens patients with low immunity. However, the number of antifungal drugs on the market is limited in comparison to the number of available antibacterial drugs. This fact, coupled with the increased frequency of fungal resistance, makes it necessary to develop new therapeutic strategies. Combination drug therapy is one of the most widely used and effective strategy to alleviate this problem. In this paper, we were aimed to evaluate the combined antifungal effects of four CCBs (calcium channel blockers), amlodipine (AML), nifedipine (NIF), benidipine (BEN) and flunarizine (FNZ) with fluconazole against *C*. *albicans* by checkerboard and time-killing method. In addition, we determined gene (*CCH1*, *MID1*, *CNA1*, *CNB1*, *YVC1*, *CDR1*, *CDR2* and *MDR1*) expression by quantitative PCR and investigated the efflux pump activity of resistant *candida albicans* by rhodamine 6G assay to reveal the potential mechanisms. Finally, we concluded that there was a synergy when fluconazole combined with the four tested CCBs against resistant strains, with fractional inhibitory concentration index (FICI) <0.5, but no interaction against sensitive strains (FICI = 0.56 ~ 2). The mechanism studies revealed that fluconazole plus amlodipine caused down-regulating of *CNA1*, *CNB1* (encoding calcineurin) and *YVC1* (encoding calcium channel protein in vacuole membrane).

## 1. Introduction

Candidiasis is one of the most important human opportunistic fungal infections, which threatens peoples living with immune system compromise, including solid organ transplant, AIDS or cancer patients, and hematopoietic stem cell transplant recipients. *Candida albicans* remains the most common etiological agent of candidiasis [1,2]. Fluconazole (FLC) which belongs to azole antifungals, is the mainstay in prevention and treatment of *Candida albicans* infections due to the great efficacy, reduced toxicity and inexpensive [3]. However, with the wide-spread use of FLC, the incidence of FLC-resistance or cross-resistance to multiple azole derivatives emerged [4–6]. Despite the availability of several other classes of antifungal drugs (Polyenes, Pyrimidines, Echinocandins), inherent toxicity, cost of care, and emergence of resistance remain major problems in clinic, and the available antifungal agents are still limited [7]. These problems point to an urgent and unmet need for the development of new antifungal agents or searching for new antifungal approaches. Unfortunately, the pace of developing new antifungal drugs has been extremely slow. Therefore, more and more attentions have been paid to drug combination, of which, the combination of antifungals with non-antifungals was focused on [8,9].

Calcium channel blockers (CCBs), which are commonly used drugs in treatment of cardiovascular disease, were demonstrated to have antifungal activity against *Candida albicans* or *Aspergillus fumigates* [10–12]. The synergistic antifungal properties in combination with some known antifungal agents (such as fluconazole, ketoconazole, itraconazole) have also been reported [13–16]. In addition to inhibition of fungal tube or biofilm formation, the established antifungal mechanisms were identified as targeting calcium channel protein Cch1-Mid1 in membrane and disturbing calcium homeostasis. Recently, more and more reports documented that calcium and some components in calcium-signaling pathway were related to fungal resistance and virulence [17,18]. For example, calcineurin is involved in hyphal growth, drug tolerance, virulence and stress responses in serum of some candidas [19–22], vacuolar acidification contributed to the ability of *Candida albicans* to form hyphae and establish infection [23], and that Yvc1p, the putative vacuolar calcium channel, plays an important role in *Candida albicans* infection and survival in host tissues as it is associated with fungal stress response, morphogenesis, and polarized growth [24]. Thus, some non-antifungal agents with action of disturbing calcium homeostasis may become the potential candidates to combine with FLC to fight against fungal infections [11,12,15,25,26].

In this study, we first evaluated the in Vitro activity of four clinic-commonly used CCBs, amlodipne (AML), nifedipien (NIF), benidipine (BEN), flunarizine (FNZ) combined with FLC against resistant *Candida albicans* (CA10, CA16) and sensitive *Candida albicans* (CA14, CA129). In addition, we performed time-killing curves to investigate the antifungal effects of the four CCBs combined with FLC against resistant *Candida albicans* (CA10) in different time points dynamically. Since some CCBs could disturb the calcium homeostasis by interfering with Cch1-Mid1 protein, which is a homologue of mammalian voltage-gated Ca^2+^ channels (VGCCs) [27], we determined the gene expression of *CCH1*, *MID1*, *CNA1*, *CNB1* and *YVC1*, which are closely related to the regulation of calcium concentration by real-time quantitative PCR (RT-PCR). In order to identify whether the combinations could also reverse one of the most common resistant mechanism of increased drug efflux, we conducted rhodamine 6G assays to investigate activity of efflux pump protein and RT-PCR to determine gene expression of *CDR1*, *CDR2* and *MDR1* [28].

## 2. Materials and Methods

### 2.1 Strains and media

The following *Candida albicans* strains were used in this study: the FLC resistant isolates (CA10, CA16) and FLC susceptible isolates (CA14, CA129) and the quality control strain (ATCC10231). The strains were refreshed from the frozen storage at -80°C and subcultured on the yeast–peptone–dextrose (YPD) solid medium (1% yeast extract, 2% peptone, 2% glucose and 2% agar) at least twice at 35°C before each experiment to ensure viability and purity. RPMI1640 was used as liquid medium to dilute drugs and strains.

FLC was provided by Cheng Chuang Pharmaceutical and a stock solution of 2560 μg ml^-1^ was prepared in distilled water. AML, NIF, BEN and FNZ were purchased from Chinese medicine institute and stock solutions of 2560 μg ml^-1^ were prepared in dimethyl sulphoxide (DMSO). All stock solutions were stored at -20°C.

### 2.2 MIC testing in broth microdilution assays

The determination of FLC MIC and the investigation of the interaction of tested CCBs with FLC by way of checkerboard tests on 96-well plates were performed according to the approved CLSI standard reference method for broth dilution antifungal susceptibility testing of yeasts M27-A3 [29]. For the checkerboard assays [30], 50 μl of RPMI-1640 medium containing FLC with concentration ranging from 0.125~64 μg ml^-1^, was added to the wells in the second to eleventh columns of the microtitre plate, and 50 μl of RPMI-1640 medium containing AML (or NIF, BEN and FNZ) with concentration ranging from 32~0.5 μg ml^-1^ was added to the wells in A to G line of the 96-well plate. Then, 100 μl of *Candida albicans* cell suspensions (0.5~ 2.5×10^3^ cells/ml) was added to each well mentioned above. All the wells on the plate were filled with RPMI-1640 to a final volume of 200 μl. Thus, the well H1 (the intersection of row H and column 1) was drug free that served as the growth control and wells of the twelfth column filled with only RPMI-1640 medium served as the negative control. The plate was covered with its lid, sealed with parafilm and incubated at 35°C for 24 h [31]. Readings were performed by both visual reading and optical density (OD) by determining the absorbance at 490 nm on a microplate reader. MIC endpoints were defined as the lowest concentration of drugs causing 80% decrease in viability compared to the drug-free control (MIC_80_). All experiments were performed in triplicate. Drug interactions were interpreted by fractional inhibitory concentration index (FICI) model and the percentage of growth difference (ΔE) model, respectively. The FICI was calculated as the sum of the FICs of either drug (MIC_FLC + CCB_ /MIC_FLC_ + MIC _CCB + FLC_ /MIC_CCB_). The FICI≤ 0.5 represents synergy, an FICI > 4 antagonism, and a 0.5<FICI ≤4 no interaction [32]. The ΔE model was defined by the following equation: ΔE = E _predicted_—E _measured_, with E _measured_ obtained directly from the experimental data. The following equation was derived: E _predicted_ = E_A_×E_B_, where E_A_ and E_B_ are the experimental percentages of fungal growth of each drug action alone, respectively. Statistically significant interactions of <100% were considered weak, those of 100–200% were considered moderate, and those of >200% were considered strong, as described previously [33].

### 2.3 Time-killing curves

Inoculum of (0.5 ~ 2.5) × 10^3^ cells/ml of *Candida albicans* (CA10) was used in this experiment. The final concentration was 1μg ml^-1^ for FLC when combined with AML (8 μg ml^-1^) and NIF (8 μg ml^-1^), and 2μg ml^-1^ for FLC when combined with BEN (16 μg ml^-1^) and FNZ (16 μg ml^-1^). A drug-free sample served as a growth control. The XTT [2,3-bis-(2-methoxy-4-nitro-5- sulfophenyl)-2H-tetrazolium-5-carboxanilide] test was performed to detect the cell viability after different treatments according to the method described previously [30,34]. Briefly, at predetermined time points (0, 6, 12, 24, and 48 h after incubation at 35°C), 100 μl aliquot from each treatment mixture was transferred to a well of new 96-well microtitre plate, and then a 100 μl aliquot of XTT-menadione solution was added (Prior to each assay, XTT purchased from Sigma was dissolved in a saturated solution at 0.5 μg ml^-1^ in Ringer’s lactate. The solution was filter sterilized through a 0.22 μm filter, and then 100 mM menadione in acetone was added to a final concentration of 10 μM). The plate was then incubated in the dark for up to 2 h at 35°C. After that, XTT reduction was assessed by determining the absorbance at 490 nm on a microplate reader (SPECTRA MAX190, Thermo lab systems, USA). All experiments were conducted in triplicate, and the results were reported as mean values. Thus, growth- and metabolism-inhibitory effects of the drugs alone and in combination were observed based on the results of spectrophotometric methods.

### 2.4 Real-time quantitative PCR

For determining the expression levels of calcium-regulation related genes *CCH1*, *MID1*, *CNA1*, *CNB1*, *YVC1*, and genes encoding membrane transport protein (*CDR1*, *CDR2* and *MDR1*), *Candida albicans* (CA10) cells were grown to mid-log phase in RPMI-1640 medium at 35°C after treatment with drugs alone or in combination at the following final concentrations: FLC at 1 μg ml^-1^; AML at 8 μg ml^-1^. Cultures without drugs served as the control. Cells were then harvested for RNA extraction. Cell total RNA was isolated using the hot phenol method described as previous [35]. Then, diluted RNA was treated with First-Stand cDNA SynthesisSuperMix kit (TransGen Biotech) and was reversely transcribed at 42°C for 30 min and 85°C for 5 min according to the manufacturer’s instructions. RT-PCR reactions were mixed with RNA, Ultro SYBR Mixture (with ROX) (Transgene, Beijing, China) and gene primers (The sequences of the primers are listed in Table 1) in triplicate. The *ACT* gene was used as the endogenous control. The RT-PCR was carried out with an ABI ViiA 7 (Applied Biosystems) sequence detection system using SYBR Green I (Cwbiotech) in duplicate for three separate experiments. An aliquot of 25 ml PCR mix was used for each gene and the cycling conditions were 95°C for 10 min, followed by 40 cycles of 95°C for 15 s and 60°C for 1 min.

**Table 1:** Gene-specific primers used for RealTime-PCR.

Gene	Primer sequences (5’→3’)	Product length (base pairs)
ACT	F:GTT AGG TCTA AAG TCG AAG TCA TC	516
	R:GTT TGG TCA ATA CCA GCA GCT TCC AAA	
CCH1	F:AGC ATG TAA GAT AGC CAT CCC G	213
	R:TAT GCC GCT GGT TCT CCA TT	
MID1	F: ACA CCA ATA AGA GAC ACA ATC ATT C	281
	R:GTG GTG GTG GTC TCG GTT AAT	
CNA1	F:GCC AAC GAA GAA GAG AAG GC	189
	R:ACC TTT GGG TAA TGA TCC CCG	
CNB1	F:CAA AAA TGG GGG CTA ACG CA	127
	R:TCA ATT TGC CCT GAC CCA TCT	
YVC1	F:TCA ATT TTT GGC GAG GAC GC	203
	R:TCC TTG CCG TCA CCT TTA CC	

### 2.5 Rhodamine 6G efflux assay

To identify whether efflux pump function could be affected by these four CCBs, the functional activity of drug efflux pumps was tested. Briefly [36], cells were shaken overnight at 35°C in YPD broth, transferred to fresh YPD broth and incubated at 35°C for 4 h. The cells were harvested by centrifuging and adjusted to 1× 10^7^ Cells/ml in YPD broth. A final concentration of 10 μmol Rh6G was added to the cell suspension. *Candida albicans* (CA10) cells grown to mid-log phase in RPMI-1640 medium at 35°C were collected by centrifuging, washed and resuspended in PBS. The cells were then de-energized for 2 h in PBS (without glucose). The de-energized cells were pelleted, washed three times with PBS and resuspended with PBS containing 5% glucose. Energy dependent efflux was measured after the addition of tested CCBs to the cells. Samples, 500 μl each, were withdrawn at specific time intervals (5, 10, 15, 20, 25, 30 min) and the fluorescence of the reaction mixture was recorded using a BD FACScalibur flow cytometer (Becton Dickinson) with excitation at 485 nm and emission at 538 nm, respectively. Rh6G-free and Rh6G-alone groups as controls were included in all the experiments.

### 2.6 Statistical analysis

Data were analyzed using SPSS Statistics software with Student’s t-test. Values presented as means ± standard deviation of three replicates, * P <0.01 indicated the levels of significant difference (at least 2.0-fold compared to the control group).

## 3. Results and Discussion

### 3.1 Calcium channel blockers tested significantly increased the sensitivity of fluconazole to resistant *Candida albicans*

**Fig 1:**
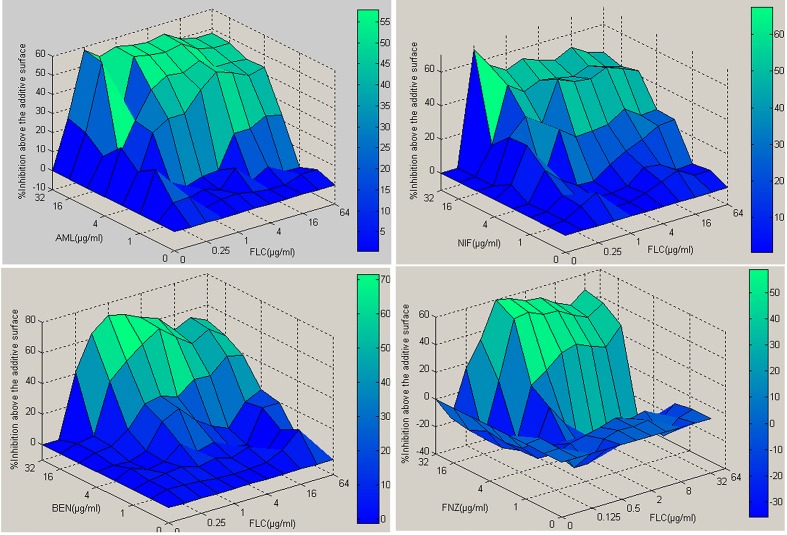
Three-dimensional plots of fluconazole combined with tested calcium channel blockers against CA10 (resistant) using the MATLAB program. The ΔE values obtained for each combination can be depicted on the z axis to construct a 3D surface plot. Peaks above and below the 0 plane indicate synergistic and antagonistic combinations, respectively. The 0 plane indicates the absence of SS interaction. The color-coding on the right indicates that the closer to red in the top of the bar, the more effective the drug combinations.

Using the CLSI standard reference method (M27-A3) for broth dilution antifungal susceptibility testing of yeasts [29], the MIC_80_ for fluconazole of sensitive strains CA14, CA129 were 0.5 μg ml^-1^ and 2 μg ml^-1^, of resistant strains (CA10, CA16) were all 512 μg ml^-1^. Whereas the MIC for CCBs (AML, NIF, BEN, FNZ) of all the tested strains were >512 μg ml^-1^, exhibiting no antifungal activity. However, the MIC_80_ of FLC for resistant strains were decreased to 1~2 μg ml^-1^ in the presence of AML (8 μg ml^-1^), NIF (8 μg ml^-1^), BEN (16 μg ml^-1^), FNZ (16 μg ml^-1^). Calculation of the fractional inhibitory concentration index (see Materials and Methods) produced values of 0.017 ~ 0.035, indicating significant synergism (Table 2 and S1 Table). The results interpreted by ΔE method also demonstrated to be strong synergistic with very high percentages of interactions ranging from 964.71% to 1866.19% (Fig 1).

**Table 2:** The combined antifungal effects of fluconazole with calcium channel blockers against resistant *Candida albicans* evaluated by FICI.

		MICs (μg ml^-1^)		Interpretation	
Drugs	Strains	C_A_	C_B_	FICI	IN
FLC+AML	CA10	1	8	0.017	SYN
	CA16	1	8	0.017	SYN
FLC+NIF	CA10	2	8	0.019	SYN
	CA16	2	8	0.019	SYN
FLC+BEN	CA10	2	16	0.035	SYN
	CA16	2	16	0.035	SYN
FLC+FNZ	CA10	1	16	0.033	SYN
	CA16	1	16	0.033	SYN

All data are the averages of triplicate experiments. The minimal inhibitory concentration of Fluconazole and calcium channel blockers when used alone were all >512 μg ml^-1^. Abbreviation: MIC, Minimal inhibitory concentration; FLC, Fluconazole; AML, Amlodipine; NIF, Nifedipine; BEN, Benidipine; FNZ, Flunarizine; C_A_, the minimal inhitory concentration of fluconazole when used in combination with calcium channel blocker; C_B_, the minimal inhitory concentration of calcium channel blocker when used in combination with fluconazole; IN, interpretation; SYN, synergism.

For sensitive strains, the MIC_80_ of FLC showed almost no change when combined with these four CCBs compared with FLC alone with FICI ranging from 0.56 to 2 and the ΔE method showed very low percentages of synergistic interaction, indicating no interaction between fluconazole and AML (NIF, BEN, FNZ) (data not shown).These observations indicate that tolerance to fluconazole of resistant *Candida albicans* is lost in the presence of the four CCBs and that AML proved to be the best candidate for combination with FLC against resistant *Candida albicans*.

### 3.2 Time-kill curves

**Fig 2:**
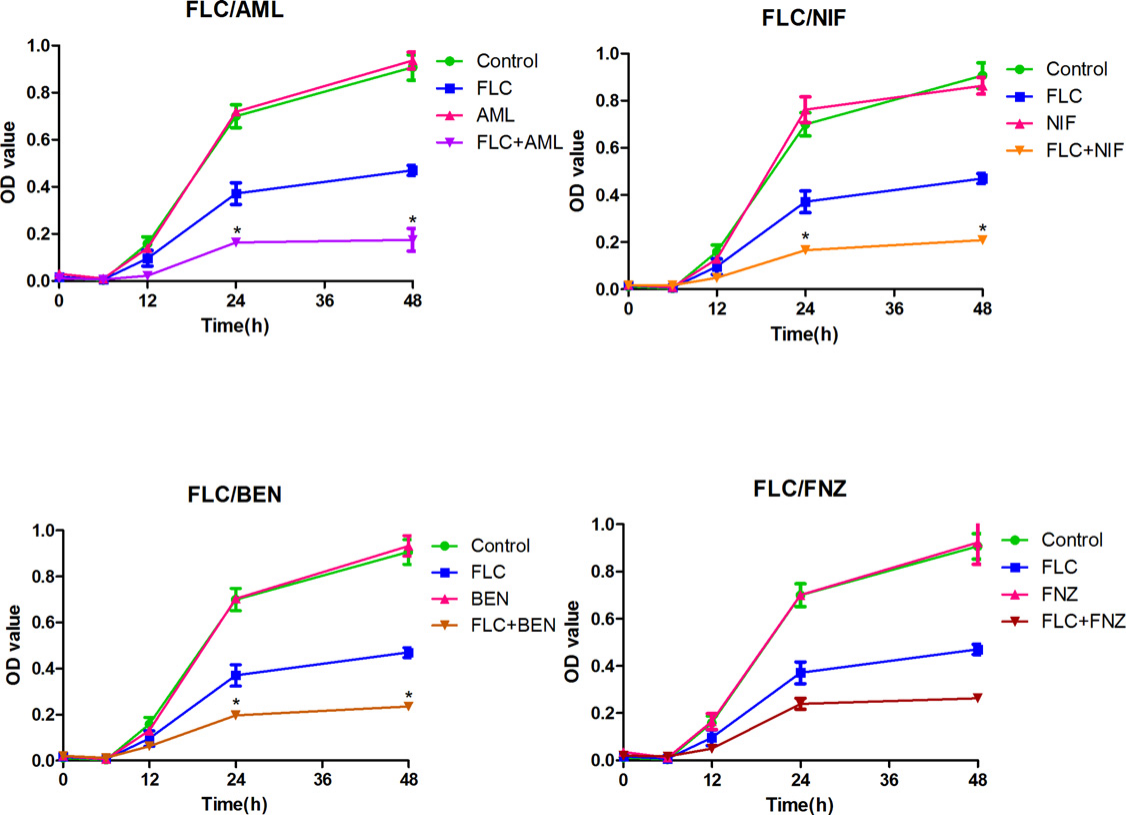
The Time-kill curves of fluconazole in combination with tested calcium channel blockers against resistant *Candida albicans* (CA10). Cells were diluted in RPMI 1640 medium containing FLC, AML, NIF, BEN, FNZ and combination of FLC with the four CCBs. The concentration of FLC was 1 μg ml^-1^ when combined with AML (8 μg ml^-1^) and FNZ (16 μg ml^-1^), and the concentration of FLC was 2 μg ml^-1^ when combined with NIF (8 μg ml^-1^) and BEN (16 μg ml^-1^). Values represent the means ±standard deviation of three replicates. * P < 0.01 and at least 2.0-fold compared to the fluconazole group.

The dynamic combined antifungal effects of FLC with CCBs (AML, NIF, BEN, FNZ) against resistant *Candida albicans* (CA10) were confirmed by time-kill studies. The intensity and nature of interactions between FLC and the four CCBs were depicted by time-kill curves with OD value obtained from the XTT reduction assay as Y-axis and time as X-axis (Fig 2 and S1 Fig). The results showed little difference between groups containing drugs and control group in the first 6 h. While, a growth delay exhibited in groups containing FLC from the 6^th^ hours, and that drug combination groups showed more obvious growth delay. At 24^th^ and 48^th^, the OD value was reduced more than two-fold in the groups of FLC combined with AML, NIF and BEN compared with FLC alone group, indicating a synergism [37]. Although the OD values were not significant different between FLC/FNZ combination group and FLC alone group, the results showed an apparent growth delay in FLC/FNZ combination group compare with FLC alone group. Among these four combinations, FLC/AML group showed the best antifungal effects against CA10 as times goes on, and FLC/NIF group showed weaker antifungal effects compared with FLC/AML. The results demonstrated that AML, NIF, BEN and FNZ could eliminate FLC tolerance against resistant *Candida albicans*, which was consistent with those from the checkerboard microdilution assays.

### 3.3 Fluconazole combined with amlodipine down-regulated the expression of *CNA1*, *CNB1* and *YVC1*

**Fig 3:**
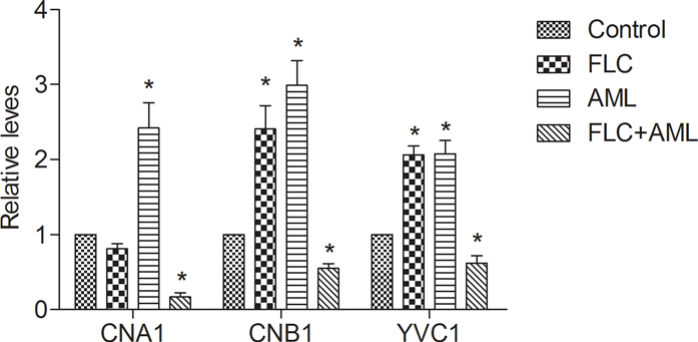
Relative expression of *CNA1*, *CNB1* and *YVC1* following treatment with fluconazole (FLC) and amlodipine (AML) alone or in combination in CA10. Cells were treated with fluconazole at 1 μg ml^-1^, amlodipine at 16 μg ml^-1^ alone or in combination. Total RNA was extracted and reversely transcribed to cDNA. cDNA was then used for real-time quantitative PCR to detect expression levels of *CNA1*, *CNB1* and *YVC1*. Values represent the means ± standard deviation of three replicates. * P < 0.01 and at least 2.0-fold compared to the control group.

The results of RT-PCR assays showed that AML alone caused almost three-fold up-regulation in the level of *CNA1* and *CNB1* transcription and the expression of *CNB1* was increase on FLC challenge by two-fold compared with the control group (P <0.01). In contrast, the combination of FLC and AML significantly down-regulated the *CNA1* and *CNB1* compared with AML challenge alone by a greater than almost ten-fold and three-fold, respectively (P < 0.01). The expression of *CNB1* was up-regulated on FLC challenge compared with the control group by two-fold (P < 0.01). For the expression level of *YVC1*, it was increased almost two-fold (P < 0.01) after the treatment of FLC or AML alone, while the drug combination two-fold down-regulated its expression compared with the two drugs alone (P <0.01) (Fig 3 and S2 Fig). What was contrary to the nature mechanisms of CCBs, there was no significant difference in the expression level of *CCH1* and *MID1* among drugs alone group, drugs combination group and control group (data not shown). As calcineurin is an important enzyme in regulating calcium homeostasis in fungal cells [38,39], and YVC1p mainly transports excess calcium in cytosol to vacuole [24,40], we speculated that: ①the treatment of FLC combined with AML disrupted calcium concentration in CA10. The results may cause an increase of calcium concentration; ② the combination inhibited the expression *CNA1*, *CNB1* and *YVC1*, thus restricted calcineurin and YVC1p to exert their functions in calcium regulation in fungal cells; ③ the increased calcium concentration in fungal cells inhibited the growth of *Candida albicans* and eliminated FLC tolerance to resistant *Candida albicans*. However, how might the calcium concentration change after drugs exposure, and if the drug combination caused an increase of calcium concentration, what was the source of calcium in *Candida albicans* cells? These all remain mysterious and need us to do a further research.

### 3.4 There was no significant relationship of decrease of drug efflux and synergism of fluconazole plus amlodipine against resistant *Candida albicans*

**Fig 4:**
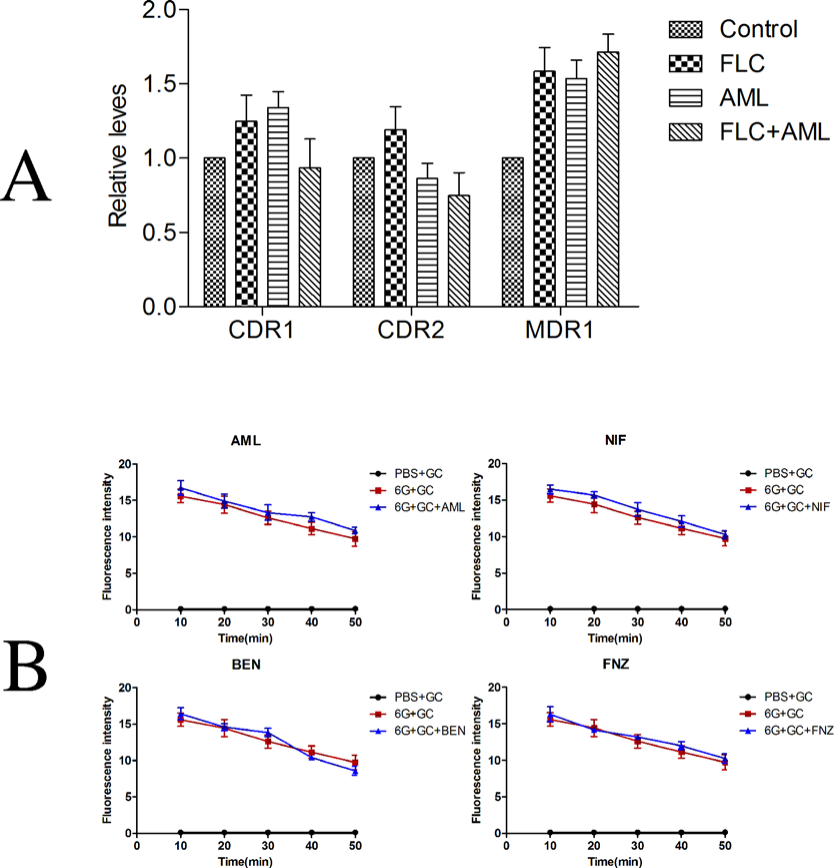
The influence of tested calcium channel blockers on fluconazole efflux. (A). Relative expression of *CDR1*, *CDR1* and *MDR1* following treatment with fluconazole (FLC) and amlodipine (AML) alone or in combination in CA10. Cells were treated with fluconazole at 1 μg ml^-1^, amlodipine at 16 μg ml^-1^ alone or in combination. Total RNA was extracted and reversely transcribed to cDNA. cDNA was then used for real-time quantitative PCR to detect expression levels of *CDR1*, *CDR1* and *MDR1*. Values represent the means ± standard deviation of three replicates. (B). The influence of amlodipine, nifedipine, benifdipine and flunarizine on efflux of fluconazole was tested by rhodamine 6G assay. There was almost no difference in fluorescence intensity with or without the tested calcium channel blocker. Values represent the means ± standard deviation of three replicates. * P < 0.01 and at least 2.0-fold compared to the control group.

The resistance of *Candida albicans* to FLC has been identified as different mechanisms, such as mutations in *ERG11* (encoding the drug target protein lanosterol 14α-demethylase) and overexpression of multidrug transporters and efflux pump-encoding genes *CDR1*, *CDR2*, and *MDR1* [5,41]. In the purpose of testing whether the presence of tested CCBs could also reverse the increase of drug efflux, we investigated the activity of efflux pumps by Rhodamine 6G assay and determined the expression changes of drug resistant genes *CDR1*, *CDR2* and *MDR1* by RT-PCR. The results demonstrated a stress increase in the expression level of *CDR1*, *CDR2* and *MDR1* after treatment of FLC alone compared with control group. The combined group down-regulated the expression level of *CDR1* and *CDR2* compared with drug alone groups, but there was no significant differences (Fig 4A and S3A Fig). For the expression level of *MDR1*, there was almost no difference between the drugs alone group and combined group. The results of Rhodamine 6G efflux assay indicated that when cells were resuspended in PBS containing 5% glucose could induce an ATP-driven drug efflux, FLC-resistant *Candida albicans* pumped out Rh6G. However, the addition of tested CCBs did not inhibit the efflux of Rh6G (Fig 4B and S3B Fig). Taken together, the results of Rhodamine 6G assay and RT-PCR method demonstrated that the synergistic antifungal effects of FLC combined with tested CCBs was irrelevant to reverse the mechanism of drug efflux.

## Supporting Information

S1 Fig The data for time-kill curves. Cells were diluted in RPMI 1640 medium containing FLC, AML, NIF, BEN, FNZ and combination of FLC with the four CCBs. The concentration of FLC was 1 μg ml^-1^ when combined with AML (8 μg ml^-1^) and FNZ (16 μg ml^-1^), and the concentration of FLC was 2 μg ml^-1^ when combined with NIF (8 μg ml^-1^) and BEN (16 μg ml^-1^). The OD value was read in triplicate.
(DOC)

S2 Fig The data for relative expression of *CNA1*, *CNB1* and *YVC1*. Cells were treated with fluconazole at 1 μg ml^-1^, amlodipine at 16 μg ml^-1^ alone or in combination. Total RNA was extracted and reversely transcribed to cDNA. cDNA was then used for real-time quantitative PCR to detect expression levels of *CNA1*, *CNB1* and *YVC1*. The experiment was conducted in triplicate.
(DOC)

S3 Fig The data for relative expression of *CDR1*, *CDR2* and *MDR1* and fluorescence intensity of rhodamine 6G of different groups. Relative expression of *CDR1*, *CDR1* and *MDR1* following treatment with fluconazole (FLC) and amlodipine (AML) alone or in combination in CA10. Cells were treated with fluconazole at 1 μg ml^-1^, amlodipine at 16 μg ml^-1^ alone or in combination. Total RNA was extracted and reversely transcribed to cDNA. cDNA was then used for real-time quantitative PCR to detect expression levels of *CDR1*, *CDR1* and *MDR1*. The experiment was conducted in triplicate. The influence of amlodipine, nifedipine, benifdipine and flunarizine on efflux of fluconazole was tested by rhodamine 6G assay. The fluorescence intensity of rhodamine 6G were conducted in triplicate.
(DOC)

S1 Table The data for combined antifungal effects of fluconazole with calcium channel blockers against resistant Candida albicans evaluated by FICI. The growth rate of resistant *C*. *albicans* (CA10) after the interaction of FLC with the four tested calcium channel blockers were shown in table S5a, 5b, 5c and 5d respectively.
(DOC)

## References

[1] Pierce CG, Lopez-Ribot JL. Candidiasis drug discovery and development: new approaches targeting virulence for discovering and identifying new drugs. Expert Opin Drug Discov. 2013; 8: 1117–26. 10.1517/17460441.2013.807245 23738751 PMC3984500

[2] Orasch C, Marchetti O, Garbino J, Schrenzel J, Zimmerli S, Muhlethaler K, et al Candida species distribution and antifungal susceptibility testing according to European Committee on Antimicrobial Susceptibility Testing and new vs. old Clinical and Laboratory Standards Institute clinical breakpoints: a 6-year prospective candidaemia survey from the fungal infection network of Switzerland. Clin Microbiol Infect. 2014; 20: 698–705. 10.1111/1469-0691.12440 24188136

[3] Guo F, Yang Y, Kang Y, Zang B, Cui W, Qin B, et al Invasive candidiasis in intensive care units in China: a multicentre prospective observational study. J Antimicrob Chemother. 2013; 68: 1660–8. 10.1093/jac/dkt083 23543609

[4] Pfaller MA, Rhomberg PR, Messer SA, Jones RN, Castanheira M. Isavuconazole, micafungin, and 8 comparator antifungal agents' susceptibility profiles for common and uncommon opportunistic fungi collected in 2013: temporal analysis of antifungal drug resistance using CLSI species-specific clinical breakpoints and proposed epidemiological cutoff values. Diagn Microbiol Infect Dis. 2015; 82: 303–13. 10.1016/j.diagmicrobio.2015.04.008 25986029

[5] Eddouzi J, Parker JE, Vale-Silva LA, Coste A, Ischer F, Kelly S, et al Molecular mechanisms of drug resistance in clinical Candida species isolated from Tunisian hospitals. Antimicrob Agents Chemother. 2013; 57: 3182–93. 10.1128/AAC.00555-13 23629718 PMC3697321

[6] Chen TC, Chen YH, Chen YC, Lu PL. Fluconazole exposure rather than clonal spreading is correlated with the emergence of Candida glabrata with cross-resistance to triazole antifungal agents. Kaohsiung J Med Sci. 2012; 28: 306–15. 10.1016/j.kjms.2011.11.011 22632885 PMC11916436

[7] Sharma M, Biswas D, Kotwal A, Thakuria B, Kakati B, Chauhan BS, et al Ibuprofen-mediated reversal of fluconazole resistance in clinical isolates of Candida. J Clin Diagn Res. 2015; 9: DC20–2. 10.7860/JCDR/2015/10094.5494 PMC4347079 25737988

[8] Liu S, Hou Y, Chen X, Gao Y, Li H, Sun S. Combination of fluconazole with non-antifungal agents: a promising approach to cope with resistant Candida albicans infections and insight into new antifungal agent discovery. Int J Antimicrob Agents. 2014; 43: 395–402. 10.1016/j.ijantimicag.2013.12.009 24503221

[9] Azevedo MM, Teixeira-Santos R, Silva AP, Cruz L, Ricardo E, Pina-Vaz C, et al The effect of antibacterial and non-antibacterial compounds alone or associated with antifugals upon fungi. Front Microbiol. 2015; 6: 669 10.3389/fmicb.2015.00669 26191055 PMC4490243

[10] Yu Q, Xiao C, Zhang K, Jia C, Ding X, Zhang B, et al The calcium channel blocker verapamil inhibits oxidative stress response in Candida albicans. Mycopathologia. 2014; 177: 167–77. 10.1007/s11046-014-9735-7 24577794

[11] Yu Q, Ding X, Zhang B, Xu N, Jia C, Mao J, et al Inhibitory effect of verapamil on Candida albicans hyphal development, adhesion and gastrointestinal colonization. FEMS Yeast Res. 2014; 14: 633–41. 10.1111/1567-1364.12150 24650198

[12] Rodrigues AA, Pina-Vaz C, Mardh PA, Martinez-de-Oliveira J, Freitas-da-Fonseca A. Inhibition of germ tube formation by Candida albicans by local anesthetics: an effect related to ionic channel blockade. Curr Microbiol. 2000; 40: 145–8. 10679044 10.1007/s002849910030

[13] Krajewska-Kulak E, Niczyporuk WW. Anticandidal activity of flunarizine. Mater Med Pol. 1993; 25: 143–4. 8072319

[14] Afeltra J, Vitale RG, Mouton JW, Verweij PE. Potent synergistic in vitro interaction between nonantimicrobial membrane-active compounds and itraconazole against clinical isolates of Aspergillus fumigatus resistant to itraconazole. Antimicrob Agents Chemother. 2004; 48: 1335–43. 15047538 10.1128/AAC.48.4.1335-1343.2004 PMC375285

[15] Yu Q, Ding X, Xu N, Cheng X, Qian K, Zhang B, et al In vitro activity of verapamil alone and in combination with fluconazole or tunicamycin against Candida albicans biofilms. Int J Antimicrob Agents. 2013; 41: 179–82. 10.1016/j.ijantimicag.2012.10.009 23265915

[16] Bulatova NR, Darwish RM. Effect of chemosensitizers on minimum inhibitory concentrations of fluconazole in Candida albicans. Med Princ Pract. 2008; 17: 117–21. 10.1159/000112964 18287794

[17] Liu S, Hou Y, Liu W, Lu C, Wang W, Sun S. Components of the calcium-calcineurin signaling pathway in fungal cells and their potential as antifungal targets. Eukaryot Cell. 2015; 14: 324–34. 10.1128/EC.00271-14 25636321 PMC4385803

[18] Liu FF, Pu L, Zheng QQ, Zhang YW, Gao RS, Xu XS, et al Calcium signaling mediates antifungal activity of triazole drugs in the Aspergilli. Fungal Genet Biol. 2015; 81: 182–90. 10.1016/j.fgb.2014.12.005 25554700

[19] Chen YL, Yu SJ, Huang HY, Chang YL, Lehman VN, Silao FG, et al Calcineurin controls hyphal growth, virulence, and drug tolerance of Candida tropicalis. Eukaryot Cell. 2014; 13: 844–54. 10.1128/EC.00302-13 24442892 PMC4135728

[20] Zhang J, Silao FG, Bigol UG, Bungay AA, Nicolas MG, Heitman J, et al Calcineurin is required for pseudohyphal growth, virulence, and drug resistance in Candida lusitaniae. PLoS One. 2012; 7: e44192 10.1371/journal.pone.0044192 22952924 PMC3432075

[21] Blankenship JR, Heitman J. Calcineurin is required for Candida albicans to survive calcium stress in serum. Infect Immun. 2005; 73: 5767–74. 16113294 10.1128/IAI.73.9.5767-5774.2005 PMC1231066

[22] Chen YL, Brand A, Morrison EL, Silao FG, Bigol UG, Malbas FF Jr., et al Calcineurin controls drug tolerance, hyphal growth, and virulence in Candida dubliniensis. Eukaryot Cell. 2011; 10: 803–19. 10.1128/EC.00310-10 21531874 PMC3127677

[23] Patenaude C, Zhang Y, Cormack B, Kohler J, Rao R. Essential role for vacuolar acidification in Candida albicans virulence. J Biol Chem. 2013; 288: 26256–64. 10.1074/jbc.M113.494815 23884420 PMC3764829

[24] Yu Q, Wang F, Zhao Q, Chen J, Zhang B, Ding X, et al A novel role of the vacuolar calcium channel Yvc1 in stress response, morphogenesis and pathogenicity of Candida albicans. Int J Med Microbiol. 2014; 304: 339–50. 10.1016/j.ijmm.2013.11.022 24368068

[25] da Silva CR, de Andrade Neto JB, Sidrim JJ, Angelo MR, Magalhaes HI, Cavalcanti BC, et al Synergistic effects of amiodarone and fluconazole on Candida tropicalis resistant to fluconazole. Antimicrob Agents Chemother. 2013; 57: 1691–700. 10.1128/AAC.00966-12 23357774 PMC3623355

[26] Bagar T, Bencina M. Antiarrhythmic drug amiodarone displays antifungal activity, induces irregular calcium response and intracellular acidification of Aspergillus niger—amiodarone targets calcium and pH homeostasis of A. niger. Fungal Genet Biol. 2012; 49: 779–91. 10.1016/j.fgb.2012.07.007 22906851

[27] Teng J, Goto R, Iida K, Kojima I, Iida H. Ion-channel blocker sensitivity of voltage-gated calcium-channel homologue Cch1 in Saccharomyces cerevisiae. Microbiology. 2008; 154: 3775–81. 10.1099/mic.0.2008/021089-0 19047745

[28] Golabek K, Strzelczyk JK, Owczarek A, Cuber P, Slemp-Migiel A, Wiczkowski A. Selected mechanisms of molecular resistance of Candida albicans to azole drugs. Acta Biochim Pol. 2015; 62: 247–51. 10.18388/abp.2014_940 25901298

[29] Lockhart SR, Bolden CB, Iqbal N, Kuykendall RJ. Validation of 24-hour flucytosine MIC determination by comparison with 48-hour determination by the Clinical and Laboratory Standards Institute M27-A3 broth microdilution reference method. J Clin Microbiol. 2011; 49: 4322–5. 10.1128/JCM.05479-11 22012016 PMC3232968

[30] Sun S, Li Y, Guo Q, Shi C, Yu J, Ma L. In vitro interactions between tacrolimus and azoles against Candida albicans determined by different methods. Antimicrob Agents Chemother. 2008; 52: 409–17. 18056277 10.1128/AAC.01070-07 PMC2224779

[31] Pfaller MA, Diekema DJ. Progress in antifungal susceptibility testing of Candida spp. by use of Clinical and Laboratory Standards Institute broth microdilution methods, 2010 to 2012. J Clin Microbiol. 2012; 50: 2846–56. 10.1128/JCM.00937-12 22740712 PMC3421803

[32] Odds FC. Synergy, antagonism, and what the chequerboard puts between them. J Antimicrob Chemother. 2003; 52: 1 12805255 10.1093/jac/dkg301

[33] Shi W, Chen Z, Chen X, Cao L, Liu P, Sun S. The combination of minocycline and fluconazole causes synergistic growth inhibition against Candida albicans: an in vitro interaction of antifungal and antibacterial agents. FEMS Yeast Res. 2010; 10: 885–93. 10.1111/j.1567-1364.2010.00664.x 20707818

[34] Guo Q, Sun S, Yu J, Li Y, Cao L. Synergistic activity of azoles with amiodarone against clinically resistant Candida albicans tested by chequerboard and time-kill methods. J Med Microbiol. 2008; 57: 457–62. 10.1099/jmm.0.47651-0 18349365

[35] Gao Y, Zhang C, Lu C, Liu P, Li Y, Li H, et al Synergistic effect of doxycycline and fluconazole against Candida albicans biofilms and the impact of calcium channel blockers. FEMS Yeast Res. 2013; 13: 453–62. 10.1111/1567-1364.12048 23577622

[36] Sun LM, Liao K, Liang S, Yu PH, Wang DY. Synergistic activity of magnolol with azoles and its possible antifungal mechanism against Candida albicans. J Appl Microbiol. 2015; 118: 826–38. 10.1111/jam.12737 25641229

[37] Pfaller MA, Sheehan DJ, Rex JH. Determination of fungicidal activities against yeasts and molds: lessons learned from bactericidal testing and the need for standardization. Clin Microbiol Rev. 2004; 17: 268–80. 15084501 10.1128/CMR.17.2.268-280.2004 PMC387411

[38] Cui J, Kaandorp JA, Sloot PM, Lloyd CM, Filatov MV. Calcium homeostasis and signaling in yeast cells and cardiac myocytes. FEMS Yeast Res. 2009; 9: 1137–47. 10.1111/j.1567-1364.2009.00552.x 19678847

[39] Yu SJ, Chang YL, Chen YL. Calcineurin signaling: lessons from Candida species. FEMS Yeast Res. 2015; 15. 10.1093/femsyr/fov016 25878052

[40] Yu Q, Zhang B, Yang B, Chen J, Wang H, Jia C, et al Interaction among the vacuole, the mitochondria, and the oxidative stress response is governed by the transient receptor potential channel in Candida albicans. Free Radic Biol Med. 2014; 77: 152–67. 10.1016/j.freeradbiomed.2014.09.011 25308698

[41] Jensen RH, Astvad KM, Silva LV, Sanglard D, Jorgensen R, Nielsen KF, et al Stepwise emergence of azole, echinocandin and amphotericin B multidrug resistance in vivo in Candida albicans orchestrated by multiple genetic alterations. J Antimicrob Chemother. 2015; 70: 2551–5. 10.1093/jac/dkv140 26017038 PMC4553713

