# Supplementary material for: Synergistic Effect of Fluconazole and Calcium Channel Blockers against Resistant Candida albicans
Source: PLoS One. 2016 Mar 17;11(3):e0150859. doi: 10.1371/journal.pone.0150859 (PMC4795682; doi:10.1371/journal.pone.0150859)
Supplement: S1 Fig — Cells were diluted in RPMI 1640 medium containing FLC, AML, NIF, BEN, FNZ and combination of FLC with the four CCBs. The concentration of FLC was 1 μg ml-1 when combined with AML (8 μg ml-1) and FNZ (16 μg ml-1), and the concentration of FLC was 2 μg ml-1 when combined with NIF (8 μg ml-1) and BEN (16 μg ml-1). The OD value was read in triplicate. (DOC) [file pone.0150859.s001.doc]

S1 Fig . The data for time-kill curves

| The OD value of different groups for the first time | | | | | | | | | | |
| --- | --- | --- | --- | --- | --- | --- | --- | --- | --- | --- |
| Time (h) | Control | FLC | AML | NIF | BEN | FNZ | FLC+AML | FLC+NIF | FLC+BEN | FLC+FNZ |
| 0.0000 | 0.0089 | 0.0150 | 0.0203 | 0.0156 | 0.0166 | 0.0362 | 0.0157 | 0.0213 | 0.0402 | 0.0229 |
| 6.0000 | 0.0057 | 0.0065 | 0.0075 | 0.0024 | 0.0069 | 0.0134 | 0.0043 | 0.0123 | 0.0090 | 0.0143 |
| 12.0000 | 0.1928 | 0.0690 | 0.1337 | 0.1357 | 0.1333 | 0.2034 | 0.0223 | 0.0403 | 0.0496 | 0.0460 |
| 24.0000 | 0.6504 | 0.3196 | 0.7316 | 0.8236 | 0.6942 | 0.7172 | 0.1491 | 0.1660 | 0.1843 | 0.2199 |
| 48.0000 | 0.9590 | 0.4518 | 0.9041 | 0.8807 | 0.9540 | 0.9866 | 0.2019 | 0.2058 | 0.2347 | 0.2491 |
| The OD value of different groups for the second time | | | | | | | | | | |
| Time (h) | Control | FLC | AML | NIF | BEN | FNZ | FLC+AML | FLC+NIF | FLC+BEN | FLC+FNZ |
| 0.0000 | 0.0144 | 0.0212 | 0.0240 | 0.0204 | 0.0216 | 0.0354 | 0.0013 | 0.0231 | 0.0084 | 0.0176 |
| 6.0000 | 0.0051 | 0.0076 | 0.0088 | 0.0080 | 0.0037 | 0.0086 | 0.0050 | 0.0094 | 0.0185 | 0.0273 |
| 12.0000 | 0.1398 | 0.0863 | 0.1397 | 0.1337 | 0.1377 | 0.1528 | 0.0220 | 0.0539 | 0.0708 | 0.0466 |
| 24.0000 | 0.7014 | 0.4093 | 0.7210 | 0.7177 | 0.6985 | 0.7045 | 0.1797 | 0.1726 | 0.2011 | 0.2654 |
| 48.0000 | 0.8516 | 0.4655 | 0.9752 | 0.8863 | 0.9621 | 0.9634 | 0.2027 | 0.2149 | 0.2379 | 0.2584 |
| The OD value of different groups for the third time | | | | | | | | | | |
| Time (h) | Control | FLC | AML | NIF | BEN | FNZ | FLC+AML | FLC+NIF | FLC+BEN | FLC+FNZ |
| 0.0000 | 0.0133 | 0.0150 | 0.0478 | 0.0175 | 0.0223 | 0.0345 | 0.0167 | 0.0083 | 0.0163 | 0.0192 |
| 6.0000 | 0.0014 | 0.0072 | 0.0152 | 0.0176 | 0.0070 | 0.0115 | 0.0104 | 0.0285 | 0.0119 | 0.0100 |
| 12.0000 | 0.1429 | 0.1332 | 0.1446 | 0.1161 | 0.1194 | 0.1387 | 0.0245 | 0.0511 | 0.0693 | 0.0561 |
| 24.0000 | 0.7478 | 0.3844 | 0.7038 | 0.7439 | 0.7182 | 0.6800 | 0.1621 | 0.1608 | 0.2063 | 0.2334 |
| 48.0000 | 0.9097 | 0.4936 | 0.9289 | 0.8239 | 0.8819 | 0.8180 | 0.1194 | 0.2052 | 0.2339 | 0.2815 |

Abbreviation: FLC: fluconazole; AML, Amlodipine; NIF, Nifedipine; BEN, Benidipine; FNZ, Flunarizine
