# Supplementary material for: Synergistic Effect of Fluconazole and Calcium Channel Blockers against Resistant Candida albicans
Source: PLoS One. 2016 Mar 17;11(3):e0150859. doi: 10.1371/journal.pone.0150859 (PMC4795682; doi:10.1371/journal.pone.0150859)
Supplement: S2 Fig — Cells were treated with fluconazole at 1 μg ml-1, amlodipine at 16 μg ml-1 alone or in combination. Total RNA was extracted and reversely transcribed to cDNA. cDNA was then used for real-time quantitative PCR to detect expression levels of CNA1, CNB1 and YVC1. The experiment was conducted in triplicate. (DOC) [file pone.0150859.s002.doc]

S2 Fig . The data for relative expression of *CNA1*, *CNB1* and *YVC1*

| RT-PCR for the first time | | | | |
| --- | --- | --- | --- | --- |
|  | Control | FLC | AML | FLC+AML |
| *CNA1* | 1 | 0.791 | 2.336 | 2.125 |
| *CNB1* | 1 | 2.351 | 2.981 | 3.326 |
| *YVC1* | 1 | 2.198 | 2.26 | 1.91 |
| RT-PCR for the second | | | | |
|  | Control | FLC | AML | FLC+AML |
| *CNA1* | 1 | 0.885 | 2.789 | 2.125 |
| *CNB1* | 1 | 2.742 | 2.653 | 3.326 |
| *YVC1* | 1 | 2.001 | 2.06 | 1.91 |
| RT-PCR for the third time | | | | |
|  | Control | FLC | AML | FLC+AML |
| *CNA1* | 1 | 0.763 | 2.125 | 0.109 |
| *CNB1* | 1 | 2.119 | 3.326 | 0.626 |
| *YVC1* | 1 | 1.98 | 1.91 | 0.52 |

Abbreviation: FLC: fluconazole; AML, Amlodipine;
