# Supplementary material for: Synergistic Effect of Fluconazole and Calcium Channel Blockers against Resistant Candida albicans
Source: PLoS One. 2016 Mar 17;11(3):e0150859. doi: 10.1371/journal.pone.0150859 (PMC4795682; doi:10.1371/journal.pone.0150859)
Supplement: S3 Fig — Relative expression of CDR1, CDR1 and MDR1 following treatment with fluconazole (FLC) and amlodipine (AML) alone or in combination in CA10. Cells were treated with fluconazole at 1 μg ml-1, amlodipine at 16 μg ml-1 alone or in combination. Total RNA was extracted and reversely transcribed to cDNA. cDNA was then used for real-time quantitative PCR to detect expression levels of CDR1, CDR1 and MDR1. The experiment was conducted in triplicate. The influence of amlodipine, nifedipine, benifdipine and flunarizine on efflux of fluconazole was tested by rhodamine 6G assay. The fluorescence intensity of rhodamine 6G were conducted in triplicate. (DOC) [file pone.0150859.s003.doc]

S3A Fig. The data for relative expression of *CDR1*, *CDR2* and *MDR1*

| RT-PCR for the first time | | | | |
| --- | --- | --- | --- | --- |
|  | Control | FLC | AML | FLC+AML |
| *CDR1* | 1 | 1.09 | 1.43 | 1.12 |
| *CDR2* | 1 | 1.11 | 0.75 | 0.89 |
| *MDR1* | 1 | 1.49 | 1.67 | 1.72 |
| RT-PCR for the second | | | | |
|  | Control | FLC | AML | FLC+AML |
| *CDR1* | 1 | 1.21 | 1.37 | 0.73 |
| *CDR2* | 1 | 1.37 | 0.89 | 0.77 |
| *MDR1* | 1 | 1.77 | 1.43 | 1.83 |
| RT-PCR for the third time | | | | |
|  | Control | FLC | AML | FLC+AML |
| *CDR1* | 1 | 1.44 | 1.22 | 0.95 |
| *CDR2* | 1 | 1.09 | 0.95 | 0.58 |
| *MDR1* | 1 | 1.49 | 1.51 | 1.59 |

S3B Fig. The data for fluorescence intensity of rhodamine 6G of different groups

| The fluorescence intensity of different groups for the first time | | | | | | |
| --- | --- | --- | --- | --- | --- | --- |
| Time (min) | PBS+GC | GC+6G | GC+6G+AML | GC+6G+NIF | GC+6G+BEN | GC+6G+FNZ |
| 10 | 0.125 | 14.7 | 15.9 | 17.1 | 16.57 | 16.31 |
| 20 | 0.125 | 14.9 | 15.9 | 15.3 | 15.01 | 14.09 |
| 30 | 0.123 | 12.8 | 13.5 | 12.9 | 14.11 | 13.15 |
| 40 | 0.126 | 11.1 | 12.3 | 12.1 | 10.5 | 11.57 |
| 50 | 0.148 | 9.74 | 10.62 | 10.9 | 8.21 | 10.95 |
| The fluorescence intensity of different groups for the second time | | | | | | |
| Time (min) | PBS+GC | GC+6G | GC+6G+AML | GC+6G+NIF | GC+6G+BEN | GC+6G+FNZ |
| 10 | 0.124 | 15.6 | 17.8 | 16.4 | 15.55 | 15.29 |
| 20 | 0.123 | 15.3 | 14.8 | 15.6 | 14.59 | 14.11 |
| 30 | 0.125 | 11.6 | 14.3 | 14.7 | 13.19 | 13.19 |
| 40 | 0.123 | 10.3 | 13.4 | 11.29 | 10.29 | 12.6 |
| 50 | 0.133 | 8.69 | 10.57 | 9.8 | 8.23 | 9.87 |
| The fluorescence intensity of different groups for the third time | | | | | | |
| Time (min) | PBS+GC | GC+6G | GC+6G+AML | GC+6G+NIF | GC+6G+BEN | GC+6G+FNZ |
| 10 | 0.122 | 16.5 | 16.49 | 15.9 | 17.16 | 17.33 |
| 20 | 0.122 | 13.1 | 14 | 16.15 | 14.11 | 14.27 |
| 30 | 0.124 | 13.4 | 12.2 | 13.6 | 14.21 | 13.22 |
| 40 | 0.124 | 12 | 12.5 | 12.82 | 10.36 | 11.77 |
| 50 | 0.128 | 10.72 | 11.39 | 10.1 | 9.29 | 9.9 |

Abbreviation: GC: glucose; 6G: Rhodamine 6G; FLC: fluconazole; AML, Amlodipine; NIF, Nifedipine; BEN, Benidipine; FNZ, Flunarizine
