# Supplementary material for: Synergistic Effect of Fluconazole and Calcium Channel Blockers against Resistant Candida albicans
Source: PLoS One. 2016 Mar 17;11(3):e0150859. doi: 10.1371/journal.pone.0150859 (PMC4795682; doi:10.1371/journal.pone.0150859)
Supplement: S1 Table — The growth rate of resistant C. albicans (CA10) after the interaction of FLC with the four tested calcium channel blockers were shown in table S5a, 5b, 5c and 5d respectively. (DOC) [file pone.0150859.s004.doc]

S1a Table. The growth rate of resistant *C. albicans* CA10 after the interaction of FLC/AML

|  | | **FLC(μg/ml)** | | | | | | | | | | | |
| --- | --- | --- | --- | --- | --- | --- | --- | --- | --- | --- | --- | --- | --- |
|  |  | **0** | **0.125** | **0.25** | **0.5** | **1** | **2** | **4** | **8** | **16** | **32** | **64** | **blank** |
| **AML(μg/ml)** | **32** | 99% | 3% | 0 | 0 | 0 | 0 | 0 | 0 | 0 | 0 | 0 | 0 |
|  | **16** | 98% | 65% | 5% | 34% | 0 | 0 | 0 | 0 | 0 | 0 | 0 | 0 |
|  | **8** | 96% | 71% | 49% | 23% | 12% | 5% | 5% | 3% | 4% | 2% | 3% | 0 |
|  | **4** | 97% | 63% | 58% | 25% | 23% | 21% | 10% | 5% | 3% | 5% | 3% | 0 |
|  | **2** | 96% | 70% | 60% | 56% | 54% | 55% | 47% | 32% | 27% | 21% | 23% | 0 |
|  | **1** | 99% | 67% | 66% | 63% | 59% | 60% | 62% | 54% | 55% | 47% | 48% | 0 |
|  | **0.5** | 97% | 78% | 70% | 55% | 57% | 49% | 53% | 60% | 48% | 43% | 50% | 0 |
|  | **0** | 100% | 89% | 63% | 53% | 56% | 54% | 53% | 55% | 50% | 48% | 48% | 0 |

S1b Table. The growth rate of resistant C. albicans CA10 after the interaction of FLC/NIF

|  | | **FLC(μg/ml)** | | | | | | | | | | | |
| --- | --- | --- | --- | --- | --- | --- | --- | --- | --- | --- | --- | --- | --- |
|  |  | **0** | **0.125** | **0.25** | **0.5** | **1** | **2** | **4** | **8** | **16** | **32** | **64** | **blank** |
| **NIF(μg/ml)** | **32** | 93% | 94% | 13% | 5% | 3% | 0 | 0 | 0 | 0 | 0 | 0 | 0 |
|  | **16** | 92% | 92% | 64% | 11% | 10% | 7% | 5% | 4% | 5% | 3% | 3% | 0 |
|  | **8** | 98% | 89% | 59% | 28% | 27% | 5% | 5% | 3% | 4% | 2% | 3% | 0 |
|  | **4** | 94% | 92% | 55% | 57% | 23% | 31% | 27% | 23% | 21% | 22% | 19% | 0 |
|  | **2** | 99% | 90% | 69% | 56% | 54% | 55% | 44% | 36% | 37% | 26% | 22% | 0 |
|  | **1** | 96% | 94% | 76% | 65% | 54% | 56% | 50% | 39% | 45% | 51% | 49% | 0 |
|  | **0.5** | 98% | 97% | 79% | 52% | 56% | 60% | 50% | 43% | 44% | 54% | 41% | 0 |
|  | **0** | 100% | 91% | 87% | 65% | 57% | 58% | 54% | 51% | 56% | 50% | 47% | 0 |

S1c Table. The growth rate of resistant C. albicans CA10 after the interaction of FLC/BEN

|  | | **FLC(μg/ml)** | | | | | | | | | | | |
| --- | --- | --- | --- | --- | --- | --- | --- | --- | --- | --- | --- | --- | --- |
|  |  | **0** | **0.125** | **0.25** | **0.5** | **1** | **2** | **4** | **8** | **16** | **32** | **64** | **blank** |
| **BEN(μg/ml)** | **32** | 98% | 94% | 45% | 25% | 12% | 8% | 8% | 5% | 5% | 5% | 2% | 0 |
|  | **16** | 92% | 92% | 86% | 45% | 31% | 17% | 5% | 4% | 5% | 3% | 3% | 0 |
|  | **8** | 96% | 89% | 89% | 74% | 57% | 48% | 47% | 28% | 22% | 17% | 12% | 0 |
|  | **4** | 97% | 92% | 85% | 83% | 63% | 51% | 57% | 53% | 51% | 42% | 29% | 0 |
|  | **2** | 99% | 90% | 86% | 86% | 74% | 75% | 67% | 62% | 57% | 41% | 33% | 0 |
|  | **1** | 95% | 94% | 88% | 80% | 79% | 70% | 72% | 54% | 55% | 47% | 48% | 0 |
|  | **0.5** | 98% | 97% | 89% | 86% | 87% | 77% | 73% | 60% | 48% | 43% | 50% | 0 |
|  | **0** | 100% | 96% | 88% | 90% | 86% | 79% | 74% | 63% | 56% | 61% | 53% | 0 |

S1d Table. The growth rate of resistant C. albicans CA10 after the interaction of FLC/FNZ

|  | | **FLC(****μg/ml)** | | | | | | | | | | | |
| --- | --- | --- | --- | --- | --- | --- | --- | --- | --- | --- | --- | --- | --- |
|  |  | **0** | **0.125** | **0.25** | **0.5** | **1** | **2** | **4** | **8** | **16** | **32** | **64** | **blank** |
| **FNZ(μg/ml)** | **32** | 98% | 94% | 45% | 25% | 5% | 0 | 0 | 0 | 0 | 0 | 0 | 0 |
|  | **16** | 95% | 92% | 86% | 45% | 8% | 2 | 0 | 0 | 0 | 0 | 0 | 0 |
|  | **8** | 96% | 89% | 89% | 84% | 53% | 38% | 24% | 20% | 18% | 17% | 12% | 0 |
|  | **4** | 97% | 92% | 85% | 83% | 63% | 71% | 87% | 83% | 81% | 82% | 79% | 0 |
|  | **2** | 99% | 91% | 86% | 76% | 84% | 85% | 77% | 82% | 87% | 61% | 73% | 0 |
|  | **1** | 95% | 74% | 73% | 66% | 69% | 60% | 47% | 54% | 45% | 50% | 49% | 0 |
|  | **0.5** | 98% | 67% | 79% | 76% | 67% | 57% | 43% | 50% | 58% | 43% | 41% | 0 |
|  | **0** | 100% | 79% | 63% | 60% | 66% | 59% | 54% | 53% | 46% | 41% | 49% | 0 |

Abbreviation: FLC: fluconazole; AML, Amlodipine; NIF, Nifedipine; BEN, Benidipine; FNZ, Flunarizine
